# Surgical and chemotherapeutic experience regarding a urachal carcinoma with repeated relapse: case report and literature review

**DOI:** 10.1186/1477-7819-11-170

**Published:** 2013-08-01

**Authors:** Liang Zong, Ping Chen

**Affiliations:** 1 Department of Surgery, Su Bei People’s Hospital of JiangSu Province, Yangzhou University, Yangzhou 225001, Jiangsu Province, China

**Keywords:** Chemotherapy, Surgery, Urachal carcinoma

## Abstract

**Background:**

Urachal carcinoma is a rare tumor that is usually associated with a poor prognosis, especially the pathological type, urachal mucinous adenocarcinoma. Surgery remains the primary treatment in prolonging the overall survival time of patients.

**Case presentation:**

We report on a 41-year-old woman with urachal mucinous adenocarcinoma who underwent three surgeries and several courses of chemotherapy over a 42-month period. The first surgery, involving en-bloc excision of the urachal mass, partial urinary bladder, urachal ligament, and umbilicus was performed in May 2007. It is well known that the correct surgical scheme plays a key role in preventing recurrence or metastasis. However, a second debulking surgery with only a single salpingo-oophorectomy may have contributed directly to the patient’s subsequent left ovarian metastasis. Therefore, we strongly recommend performing a bilateral salpingo-oophorectomy once ovarian metastasis has been detected, even if the metastasis is only present on one side. Although postoperative adjuvant chemotherapy regimens, first with Taxol, carboplatin, gemcitabine, and cisplatin, and then with IFO, EPI, and mesna were consecutively administered after the first and second surgeries, they seemed less effective, since recurrence and metastasis occurred shortly after each surgical treatment. After a third debulking surgery in June 2009, docetaxel, oxaliplatin, and capecitabine were administered. This chemotherapy regimen was chosen based on an immunohistochemical test that involved the multidrug resistance gene; this test indicated that the urachal mucinous adenocarcinoma was resistant to the two chemotherapy regimens used previously. Surprisingly, the patient exhibited a marker response to the new regimen and the metastatic foci entered into a stable disease stage. However, the patient still died of diffuse metastatic disease 1.5 years later. During the whole period of treatment, we found that serum tumor markers including CA724, CA125, CA19-9, and CEA were elevated in a linear pattern, with parallel increases in line with peritoneal carcinomatosis and parallel reductions in line with response to personalized chemotherapy.

**Conclusion:**

Personalized treatment can be given to those patients who experience a poor response to initial therapy. Moreover, an immunohistochemical test for the multidrug resistance gene and serum tumor markers may supply key information in the choice of reasonable chemotherapeutics.

## Background

Urachal carcinoma is a rare form of tumor that usually originates in the bladder, and represents 0.01% of all cancers. Moreover, this lesion accounts for 0.34-0.7% of all bladder carcinomas [1]. Pathologically, a recent population-based analysis has revealed that adenocarcinoma is very common among urachal carcinomas and represents approximately 10% of all bladder adenocarcinomas [2]. The most common histological type of urachal adenocarcinoma is mucinous adenocarcinoma [3]. However, the rare variant form known as signet-ring cell carcinoma accounts for only a small proportion of the urachal mucinous adenocarcinomas. Until now, urachal adenocarcinoma has been regarded as associated with poor prognosis, especially in the case of urachal mucinous adenocarcinoma [4,5]. Surgery remains the primary treatment for prolonging the overall survival time of patients. However, the appropriate initial surgical treatment is a matter of controversy. No standard chemotherapy regimens for advanced urachal carcinoma have yet been established.

### Case presentation

**Figure 1:**
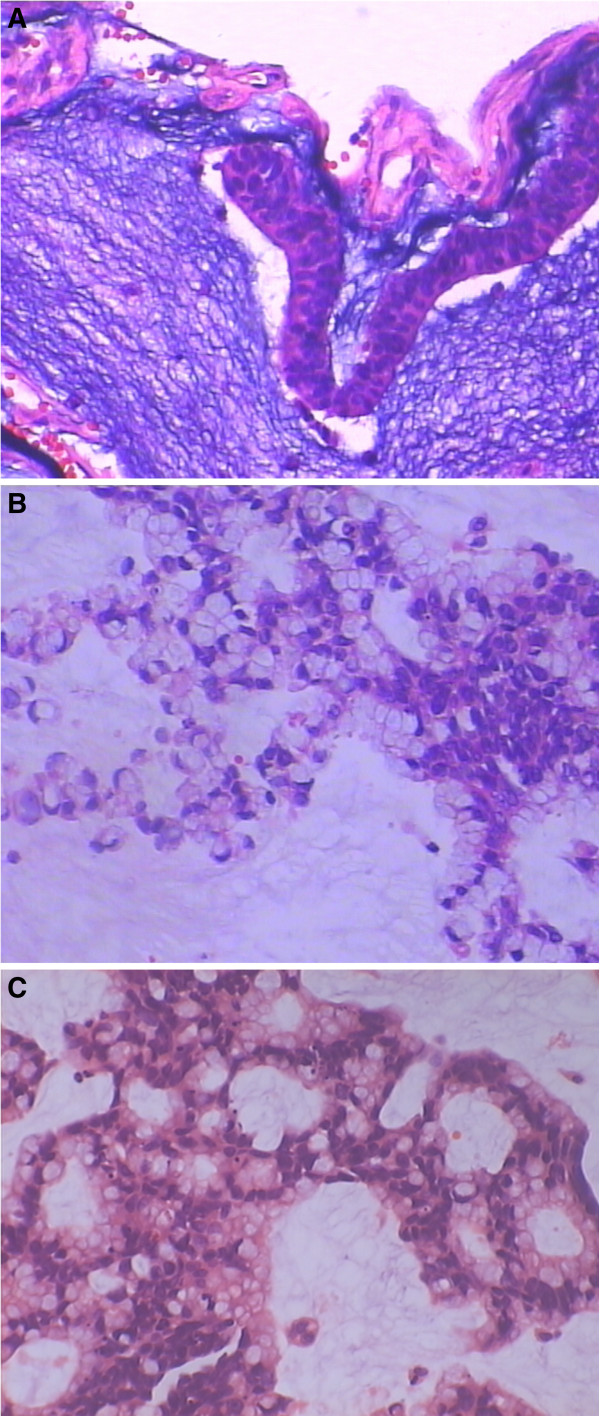
**Pathological section with hematoxylin and eosin staining after surgery. (A)** First surgery. **(B)** Second surgery. **(C)** Third surgery.

**Figure 2:**
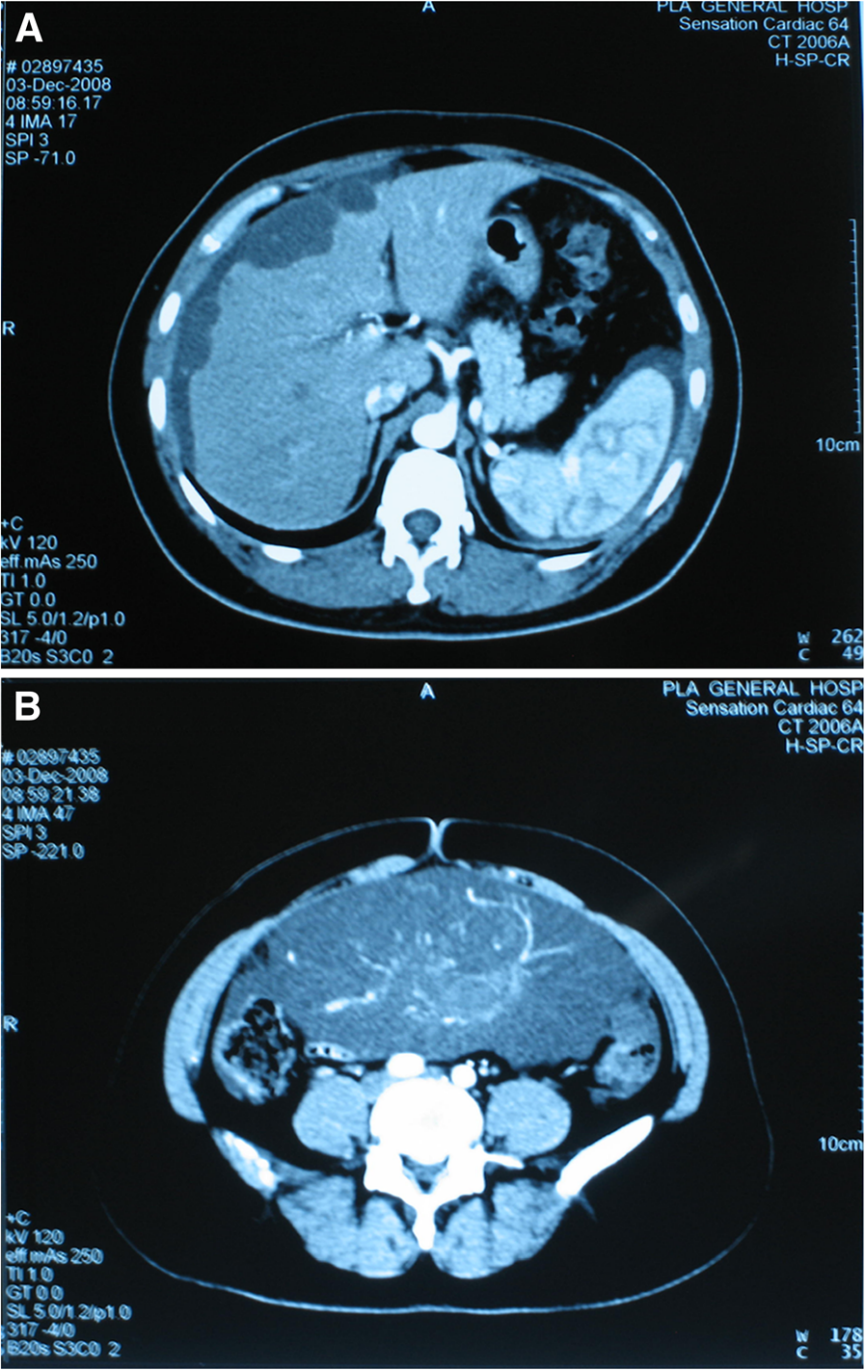
**Computed tomography. (A)** Multiple hepatic metastases. **(B)** giant local recurrent tumor infiltrating right ovary.

**Figure 3:**
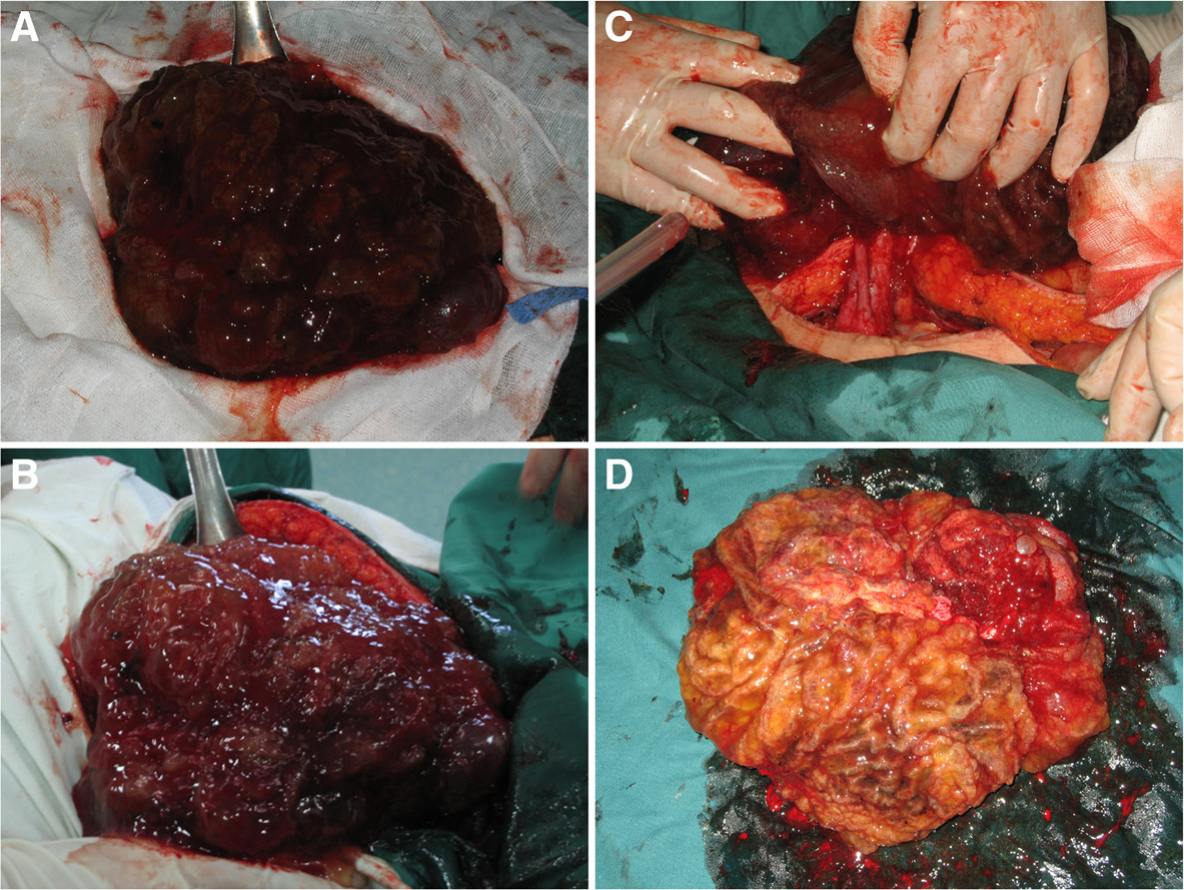
**27.0 × 17.0 cm giant recurrent tumor. (A)** A giant tumor covered by abundant mucus. **(B)** A giant tumor lying in abdominal cavity. **(C)** A giant tumor adhering to the right ovary. **(D)** A giant tumor after resection.

In June 2007, a 41-year-old woman was referred to our hospital with a palpable mass in her lower abdomen. Computed tomography showed a large mass located in the anterior pelvic cavity just superior to the bladder, but no other positive findings elsewhere. Because of the limited clinical evidence for diagnosis, laparotomy was performed for this patient. Laparotomy revealed a 12.0 × 12.0 × 9.0 cm solid lesion, which extended from the vertex of the bladder to the umbilicus. The lesion was removed en bloc together with the umbilicus, bladder dome, and adjacent peritoneum. Pathological diagnosis confirmed it to be a urachal mucinous adenocarcinoma (Figure 1A). At 1 month after diagnosis, this patient received adjuvant chemotherapy consisting of four cycles of Taxol and carboplatin. However, this regimen seemed ineffective in preventing disease progression because multiple hepatic metastases were found at only 6 months after surgery (Figure 2A). Accordingly, a revised chemotherapeutic strategy with four cycles of gemcitabine and cisplatin, as well as one cycle of interventional therapy, was administered sequentially. However, a symptom involving lower abdominal compression was gradually felt over a 1-year period. Because of this, the patient was again admitted to our hospital. Physical examination showed a large abdominal mass lying between the xiphoid process of the sternum and the umbilicus, without obvious tenderness. Abdominal computed tomography with peripheral enhancement using contrast material in the delayed phase revealed extrinsic multi-organ compression because of a giant mass, 27.0 × 17.0 cm, in the abdominal cavity (Figure 2B). At laparotomy, a giant tumor was discovered adhering to the right ovary, as well as multiple metastases in the greater omentum and liver (Figure 3). Moreover, about 300 ml of mucus was found in the pelvic cavity, suggesting seeded metastasis. We removed the tumor en bloc and resected the right ovary and greater omentum. In addition, we performed a complete dissection of the lymph node around the tumor. At the end of the surgery, we implanted a sustained-release preparation of 5-fluorouracail into the surroundings of the recurrent tumor, hepatic surface and other suspected metastatic sites in the abdominal cavity. Pathological analysis confirmed the tumor to be a recurrent carcinoma since it was located in the original tumor site (Figure 1B). Systemic chemotherapy, consisting of four cycles of IFO, mesna, and EPI, was given as a persistent treatment at 1 month after the second surgery. However, 1.5 years later, this patient was referred to our department for the third time with distension of the lower abdomen. Ultrasound detected a new mass lying in the pelvic cavity. Physical examination revealed a large abdominal mass of about 10 × 10 cm located in the lower abdomen. During laparotomy, a metastatic mass measuring 2 cm in diameter was excised. When the abdominal cavity was entered, a 10 × 10 × 8 cm solid mass was found tightly adhering to the left ovary, as well as multiple hepatic metastases. A debulking operation was performed to remove the recurrent tumor and the left ovary. During the third surgery, a sustained-release preparation of 5-fluorouracail was also implanted into the recurrent rumor site, the hepatic surface, and the bottom of the pelvic cavity. Pathological analysis supported the finding that the lesion was a stable urachal mucinous adenocarcinoma (Figure 1C). An immunohistochemical test involving the multidrug resistance gene was carried out, in which glutathione s-transferase was found to be strongly positive. Based on the detection of multidrug resistance using the gene test, four cycles of docetaxel, oxaliplatin, and capecitabine were given persistently. After the first cycle, the progression of multi-liver-foci stopped, and after the fourth cycle, 20% of these foci had disappeared. Although the docetaxel, oxaliplatin, and capecitabine regimen was efficacious in treating the tumor, temporary myelosuppression was observed, which was offset by colony-stimulating factor. However, the patient died of diffuse metastatic disease at 18 months after individualized treatment.

## Conclusion

It is sometimes difficult to differentiate between urachal and non-urachal carcinomas based only on symptoms. This is primarily because there is no specific symptom for urachal carcinoma. Furthermore, most of the tumors may develop in the submucosa or muscularis, and do not invade the mucosa of the bladder in the early stage, so symptoms are not prominent. According to the latest reports, several different imaging methods may be useful in aiding diagnosis. Ultrasound can demonstrate a tumor in the bladder dome associated with a mass containing calcification [6]. Computed tomography can identify the extent of the palpable suprapubic mass, localize calcification, and determine the involvement of local nodes. In addition, cystoscopy can reveal a tumor in the bladder dome, and concurrent bimanual examination can detect a suprapubic mass. Occasionally, biopsy may be performed via cystoscopy to confirm a diagnosis before surgery. However, for a few of the complicated cases or metastatic cases, a limited number of useful positive findings can only be made using imaging modalities; under such conditions laparotomy is necessary.

To date, the criteria for making a diagnosis of urachal carcinoma are not uniform, but clinical findings and histological evidence are both key factors. The typical criteria [1,7-9] should include: (a) a tumor in the dome of the bladder; (b) the presence of urachal residua; (c) an absence of cystitis cystica and cystitis glandularis; (d) a sharp demarcation between the tumor and the surface epithelium of the bladder; (e) the invasion of deeper muscular tissues with intact or ulcerated epithelium; (f) an extension of the tumor through the Retzius space; and (g) no evidence of a primary tumor outside of the bladder.

Our case was consistent with the typical criteria. However, for a few uncommon cases, this system is too restrictive to fulfill all the prognostic criteria. Two simpler criteria sets, which are more closely related to clinical practice, have been suggested by some scientists. The first set [4] is: (a) a tumor in the dome of the bladder; (b) the presence of urachal residua; and (c) the absence of cystitis cystica and cystitis glandularis. The second set [10] is: (a) a tumor in the dome of the bladder; (b) a sharp demarcation between the tumor and the surface epithelium of bladder; and (c) no evidence of a primary tumor outside of the bladder.

Surgical treatment plays a dominant role in the management of patients with urachal carcinoma. The achievement of a complete urachectomy including umbilectomy and negative surgical margins, and extended partial or total cystectomy, are crucial to long-term survival [1,11]. The surgical resection margin is one of the most important iatrogenic factors for prognosis, so resection must be technically feasible and frozen sections must reveal the negative resected margins [6]. If only the resection margin is clear, another important prognostic factor is tumor staging [11]. Until now, tumors have been staged using two different staging systems: the Sheldon staging system and the Mayo staging system. Both systems have predicted cancer-specific survival equally well, but we recommend the use of the Mayo staging system in future studies owing to its simplicity (Table 1). However, both systems need to be validated in future large trials.

**Table 1:** Mayo staging system for urachal carcinoma

**Stage**	**Definition**
I	Tumors confined to the urachus and or bladder
II	Tumors extending beyond the muscular layer of the urachus or the bladder
III	Tumors infiltrating the regional lymph nodes
IV	Tumors infiltrating non-regional lymph nodes or other distant sites

Late presentation of symptoms and fast progression leading to advanced tumor stage at diagnosis have resulted in a poor prognosis for urachal carcinoma, which is consequently uniformly fatal. Moreover, urachal adenocarcinomas show a tendency to local recurrence and distant metastasis after surgical treatment, most often (81%) in the first 2 years [1]. It has been revealed that postoperative local recurrence takes place earlier than distant metastasis. The most common sites of local recurrence are the pelvis, bladder, abdominal wall, and wounds. Distant metastases have been reported in a number of organs, including the lung, brain, omentum, liver, bone, and lymph nodes [1,12-14]. However, ovarian metastasis seems to be rare. In reviewing the literature, only eight cases with ovarian metastasis have been previously reported [15-22] (Table 2). Possible mechanisms of urethral recurrence are thought to include hematogenous metastasis; dissemination via the retrograde lymphatic route; and intravesical dissemination. Current research is focused on the differentiation of primary and metastatic ovarian tumors. Since the urachal adenocarcinoma metastasizes to the ovary it may mimic primary ovarian mucinous carcinoma, and this can lead to misdiagnosis. Among the eight reported cases [15-22], three were identified simultaneously with the primary urachal carcinoma and one was detected as a primary tumor before urachal adenocarcinoma was confirmed. Half of the eight cases were histologically identified as mucinous adenocarcinomas. Furthermore, frequent findings with regard to the metastatic carcinomas were bilaterality, microscopic surface involvement of epithelial cells, and an infiltrative pattern of stromal invasion. Less frequent findings that were exclusive or almost exclusive to metastatic carcinoma were a nodular invasive pattern, ovarian hilar involvement, single cell invasion, signet-ring cells, vascular invasion, and microscopic surface mucin. However, bilateral presentation of primary ovarian mucinous carcinoma is uncommon. When bilateral ovarian mucinous carcinoma is considered in the diagnosis, it is essential to exclude the possibility of metastatic carcinoma completely. The most common primary sites for metastatic ovarian mucinous carcinoma include the colon, pancreas, gallbladder, stomach, appendix, and uterine cervix [23-25]. Bladder adenocarcinoma, including urachal carcinoma, is a less common candidate for the primary tumor on these occasions.

**Table 2:** Summary of key data from eight reported cases of urachal adenocarcinoma metastatic to the ovaries

**Reference**	**Metastasis site**	**Age**	**Serum tumor marker**	**Status with primary urachal carcinoma**	**Pathology**	**Immunohistochemistry (positive or negative)**	**Treatment**	**Follow-up result**
[15]	Right ovary	50	CEA 27.9 ng/m	Simultaneous finding	Not available	-	Surgery	Not available
[16]	Both ovaries, bone, lymph nodes	50	CEA 27.9 ng/ml	Simultaneous finding	High-grade mucinous adenocarcinoma	Cytokeratin 20 and CEA, cytokeratin 7	Surgery and irradiation	Alive with disease (6 months)
[17]	Right ovary, peritoneum, sigmoid colon	54	-	Simultaneous finding	Moderately differentiated mucinous adenocarcinoma	–	Surgery and chemotherapy	Alive with disease (55 months)
[18]	Both ovaries, abdominal wall, colon mass, uterosacral ligament, bladder	64	Normal	Secondary metastasis	Borderline malignant mucinous adenocarcinoma	–	Surgery and chemotherapy	Alive with disease (3 months)
[19]	Both ovaries, bladder	39	Normal	Secondary metastasis	Well-differentiated mucinous adenocarcinoma	–	Surgery and chemotherapy	Died of disease (38 months)
[20]	Both ovaries, peritoneum, bone	26	-	Secondary metastasis	-	–	Surgery and chemotherapy	Died of disease (26 months)
[21]	Left ovary, right lung	30	-	Secondary metastasis	Well-differentiated mucinous adenocarcinoma	–	Surgery and chemotherapy	Alive without disease (11 years)
[22]	Both ovaries	72	-	Pre-finding	Moderately differentiated mucinous adenocarcinoma	–	Surgery and chemotherapy	Alive with disease (36 months)

**Figure 4:**
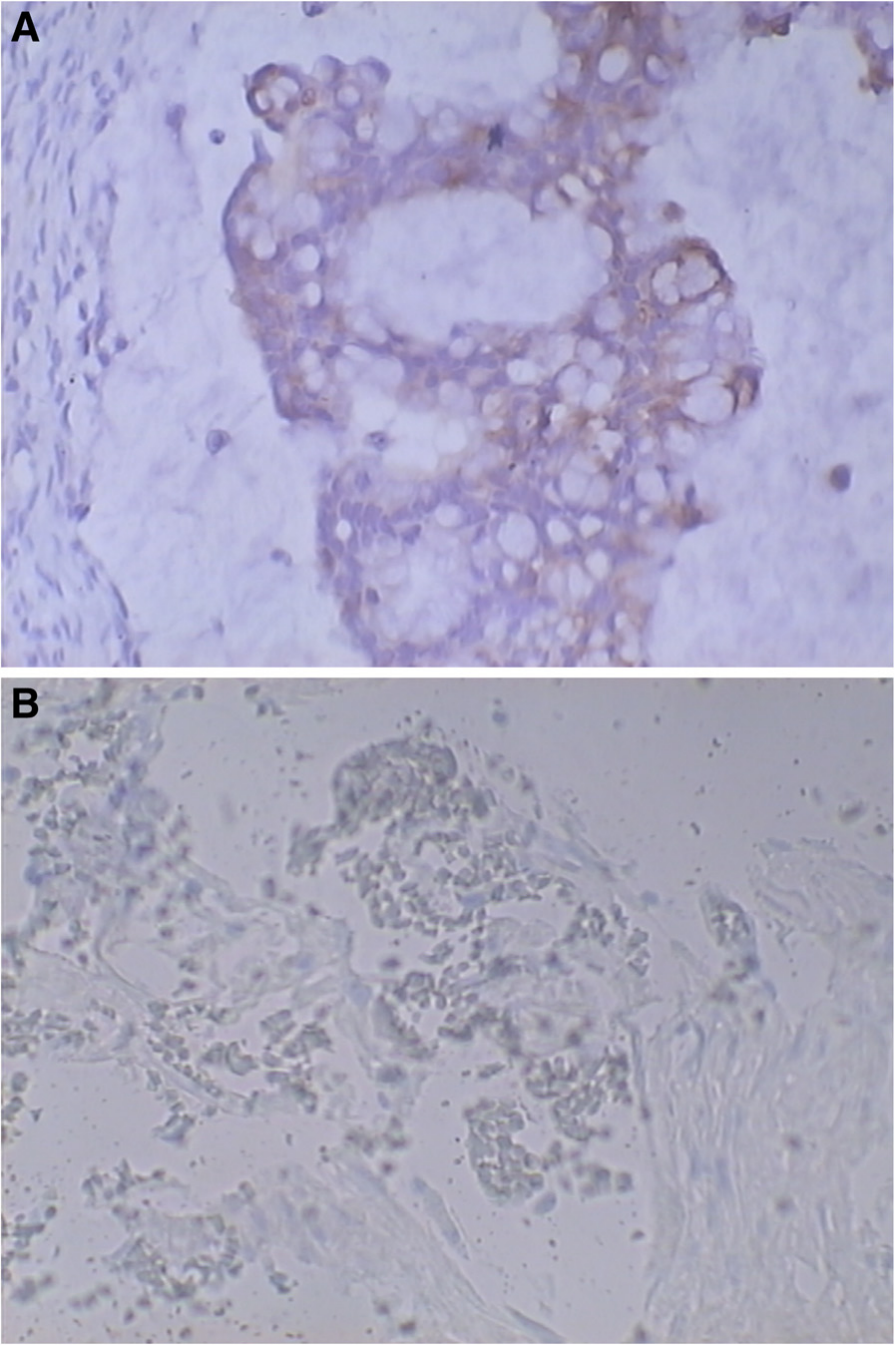
**Immunohistochemical test. (A)** Positive staining for CDX2. **(B)** Positive staining for MUC2.

Immunohistochemistry is always one important method for differentiating between primary and metastatic ovarian mucinous tumors, although there is a considerable overlap in their immunohistochemical staining patterns. In line with the latest findings, Lee [22] has recommended using a panel of CK7, CK20, CDX2, MUC2, 34βE12, and β-catenin to assist in the discrimination of urachal adenocarcinoma metastasis from primary ovarian mucinous carcinoma, and metastatic carcinoma from other organs. In Lee’s opinion [22], the coordinated expression of CK7, CK20, and CDX2 might be helpful in differentiating metastatic urachal carcinoma from primary ovarian mucinous tumor. Firstly, CK20 and CDX-2 are diffusely and strongly positive in urachal carcinoma, while about 50% of urachal carcinomas are positive for CK7, so there are two possible profiles for urachal carcinoma (CK7−/CK20+/CDX2+ vs. CK7+/CK20+/CDX2+). Secondly, CK7 − CK20+ is rarely seen in primary ovarian mucinous tumor, but CK7+/CK20+ is present in both the primary ovarian tumor and lower intestinal tract tumors. Thus, a finding of diffusely and strongly positive CDX2 plays a key role in excluding the primary ovarian tumor. In addition, because urachal carcinoma is expressed immunohistochemically as a unique colonic epithelial epitope that mimics the immunochemical profile of colonic cancer, its presence could be further confirmed by means of MUC2. Then, the only remaining issue is to discriminate a urachal origin from a lower gastrointestinal origin. However, 34βE12–/β-catenin + is rarely expressed in lower gastrointestinal tract tumors, which may help to ensure the urachal origin of metastatic ovarian tumors [9]. In this case, the immunochemical findings were partly compatible with the Lee’s diagnostic criteria [22]. CK7 and CK20 were both negative in our case, but positive CDX2 and MUC2 confirmed the tumor’s urachal origin (Figure 4). However, because only a limited number of studies with small sample size have been published on the immunohistochemical profiles of urachal carcinomas, a further large trial is need to provide more reliable data.

If suspected metastasis is encountered in the ovary, whether or not it occurs on one or both sides, it should be fully investigated and complete resection should be performed to prolong the patient’s overall survival time [26,27]. In many conditions, bilateral salpingo-oophorectomy is essential, since bilateral ovarian metastasis is the common clinical model. However, with regard to suspected single metastasis, there is still a debate on the scope of surgical resection. Kawakami *et al*. [21] reported that the use of single salpingo-oophorectomy enabled the achievement of a long survival period of more than 11 years. Conversely, Young [17] performed bilateral salpingo-oophorectomy, which was followed by early postoperative multiple metastasis at 7 months. Of course, a systematic analysis is required to obtain absolute results, including tumor stage or presence of local recurrence. Although single salpingo-oophorectomy was performed when metastasis occurred in the right ovary in our case, we support the use of bilateral salpingo-oophorectomy even for single metastasis in the ovary, especially for cases with local recurrence.

Although the role of chemotherapy and radiotherapy still remain unclear and the effects of other treatments have not been established, varied chemotherapy regimens have been reported to have some presumptive advantage [28-31] (Table 3). However, in our case, traditional chemotherapy regimens such as paclitaxel and carboplatin, 5-fluorouracail, gemcitabine, and cisplatin, and 5-fluorouracail, IFO, EPI, and mesna seemed less effective in controlling disease progression. Rapid progression of disease suggested that our patient’s carcinoma might have been chemoresistant to these chemotherapeutics. Under such an adverse condition, an individualized treatment was put forward, to achieve an improved remission. Therefore, an immunohistochemical test involving a multidrug resistance gene was carried out, in which glutathione s-transferase scored as strongly positive. This finding may explain why the initial chemotherapy regimen was less efficacious than expected. Based on this finding, a regimen of oxaliplatin, capecitabine, and docetaxel was prescribed as a salvage treatment. To our surprise, the liver metastatic foci responded well to this chemotherapy regimen and there was a 10-month remission, despite the fact that this patient experienced temporal myelosuppression, which was offset by colony-stimulating factor.

**Table 3:** Summary of response to chemotherapy in reported cases of urachal carcinoma

**Reference**	**Number of patients**	**Chemotherapy and status at last follow-up**	**Response**	**Follow-up result**
[18]	1	Paclitaxel and carboplatin	Stable	Alive with disease (3 months)
[19]	1	5-flourouracil, cisplatin, and gemcitabine	Complete response	Died of disease (38 months)
[20]	1	5-fluorouracil, folinic acid, and oxaliplatin	Complete response	Died of disease (26 months)
[28]	3	5-fluorouracil, doxorubicin, and mitomycin C	Partial response	Died of disease (12 months)
[29]	1	5-fluorouracil, mitomycin C, and mitoxantrone,	Complete response	Died of disease (28 months)
[21]	Patient 1	5-fluorouracil, doxorubicin, and cisplatin;	Complete response	Alive without disease (11 years)
		5-fluorouracil, doxorubicin, and etoposide;		
		5-fluorouracil, cisplatin, and α interferon		
[21]	Patient 2	Doxorubicin, cisplatin, and mitomycin C	Complete response	Alive without disease (10 years)
[22]	1	Docetaxel and carboplatin	Complete response	Alive with disease (36 months)
[30]	1	Tegafur, gimeracil, oteracil, and cisplatin	Complete response	Alive without disease (30 months)
[31]	1	Cisplatin, Adriamycin, vinblastine, and methotrexate	Partial response	Alive with disease (13 months)

**Figure 5:**
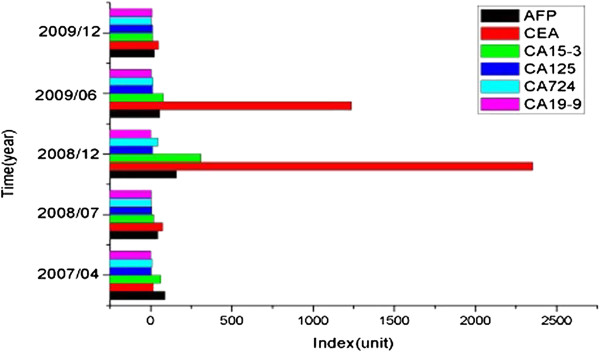
**Tumor markers changing at different follow-up dates.**

During the whole period of treatment, serum tumor markers might be one of the most important prognostic factors that can reflect the efficacy of chemotherapy and tumor recurrence. Consistent with other enteric-type adenocarcinomas, urachal adenocarcinoma might express detectable serum levels of CEA, CA125, and CA19-9 [32]. In our case, with the development of disease, these markers, including CA724, CA125, CA19-9, and CEA, were elevated in a linear pattern (Figure 5). Their levels were increased in parallel with peritoneal carcinomatosis and in parallel with a reduction in response to personal chemotherapy. Among all of the tumor markers, the most significant elevation was achieved in CA724 level prior to local recurrence and ovary metastasis (Table 4).

**Table 4:** **Laboratory findings related to tumor markers at different follow**-**up dates**

**Tumor markers**	**Index change in follow-up date**					**Normal range**
	**30 April 2007**	**8 August 2008**	**3 December 2008**	**3 June 2009**	**20 December2009**	
CA19-9	88.38 kU/l	43.80 kU/l	158.30 kU/l	57.15 kU/l	23.44 kU/l	<37.00
CA724	15.24 kU/l	73.42 kU/l	2352.0 kU/l	1237.0 kU/l	47.50 kU/l	<6.00
CA125	60.00 kU/l	19.11 kU/l	309.60 kU/l	79.20 kU/l	10.00 kU/l	<35.00
CA15-3	4.32 kU/l	5.91 kU/l	10.83 kU/l	11.56 kU/l	10.83 kU/l	<30.00
CEA	9.49 ng/ml	4.08 ng/ml	43.62 μg/ml	13.87 μg/ml	3.06 μg/ml	<5.00
AFP	2.00 ng/ml	2.55 ng/ml	2.03 μg/ml	3.39 μg/ml	7.66 μg/ml	<20.00

In conclusion, urachal carcinoma is not only a rare form of tumor, but also a difficult-to-treat disease. We support standard and radical resection to achieve a negative margin. However, even an enlarged or consecutive operation is encouraged when recurrence or peritoneal carcinomatosis occurs with a laparotomy. It is difficult to make an exact diagnosis preoperatively and a systematic review of all of the data is always essential in making a correct diagnosis based on the Mayo criteria. A standard chemotherapeutic strategy still needs to be explored in future studies, but personalized treatment can be given to those patients who experience a poor response to initial therapy. Moreover, an immunohistochemical test for multidrug resistance gene and serum tumor markers may supply key information in the choice of reasonable chemotherapeutics.

## Consent

Written informed consent was obtained from the patient for publication of this case report and accompanying images. A copy of the written consent is available for review by the editor-in-chief of this journal.

## Competing interests

Both authors declare that they have no conflicts of interest in the following areas: employment; consultancies; stock ownership; honoraria; paid expert testimony; patent applications or registrations; grants or other funding.

## Authors’ contributions

The patient was examined and operated by PC and LZ, who are responsible for the postoperative care, follow-up, and clinical information. PC examined the patient and reviewed the patient’s files. LZ performed histopathological examination. The manuscript was drafted by LZ and critically reviewed by PC. Both authors read and approved the final manuscript.
